# Supplementary material for: WordCommentsAnalyzer: A windows software tool for qualitative research
Source: F1000Res. 2018 Sep 4;7:536. Originally published 2018 May 3. [Version 2] doi: 10.12688/f1000research.14819.2 (PMC6137411; doi:10.12688/f1000research.14819.2)
Supplement: Supplementary file 3 [file f1000research-7-17614-s0002.tgz › b2a79496-4ada-47c4-9682-a99ce867836d.zip!/Eight Iranian Health Journals/Archives of Rehabilitation_59.docx]

© Archives of Rehabilitation. This work is licensed under CC BY-NC 4.0 (https://creativecommons.org/licenses/by-nc/4.0/).

Volume & Issue: دوره 6 - شماره 1

Title: بررسی عوامل خطر مؤثر بر اختلالات تکامل شیرخواران

http://rehabilitationj.uswr.ac.ir/article-1-93-fa.html

هدف: تکامل انسان یک موضوع پیچیده و بسیار عظیم است. اگر رشد را افزایش در اندازه بدن یا افزایش در بخش‌های مختلف آن بدانیم، تکامل را باید معادل تغییرات در عمل دانست که می‌تواند تحت‌تأثیر محیط کودک قرار گیرد. به‌دلیل سرعت زیاد تغییرات در دو سال اول عمر، بررسی ابعاد تکامل در سنین شیرخواری دارای اهمیت فراوان است. از طرف دیگر به‌دلیل افزایـش شیـوع اختلالات تکامل در شیرخواران پرخطر، بر آن شدیم که عوامل خطر مؤثر در اختلال تکامل شیرخواران را بررسی نماییم. روش بررسی: مطالعه به‌صورت کارآزمایی توصیفی-تحلیلی و جامعه تحقیق شامل 6150 شیرخوار 18-0 ماهه مراجعه‌کننده به درمانگاه‌های شبکه بهداشتی شهرستان کرج در طی 12 ماه، در سال‌های 1381-1380 بودند که از طریق تکمیل پرسشنامه و سپس معاینه با آزمون Infant Neurological International Battery Test برای غربالگری تأخیر تکامل توسط کارشناس کاردرمانگر در دو گروه شاهد (دارای تکامل طبیعی) و مورد (دارای تکامل غیرطبیعی) بررسی شده‌اند. یافته‌ها: نتایج نشان داد که اختلالات دستگاه اعصاب مرکزی پس از دو ماهگی از قبیل صرع، ضربه‌به‌سر و عفونت‌ها با ضریب 5/54، تشنج دوره نوزادی با ضریب 4/37، نارس‌بودن با ضریب 2/52 و عفونت‌های خون دوره نوزادی با ضریب 2/39 به‌ترتیب مهم‌ترین عوامل در پُرخطرکردن شیرخوار جهت اختلالات تکامل بعدی هستند. نتیجه‌گیری: با توجه به یافته‌های مطالعه حاضر در صورت مشکلات اقتصادی و اجرایی برای انجام غربالگری تکاملی و پیگیری تکاملی-عصبی در تمام شیرخواران در حال حاضر انجام آن در گروه شیرخواران پُرخطر کشور توصیه می‌گردد.

Title: تعیین شیوع الکتروفیزیولوژیک آناستوموز مارتین گروبر در افراد عادی

http://rehabilitationj.uswr.ac.ir/article-1-94-fa.html

هدف: ارتباط بین اعصاب مدیان و اولنار در ساعد که آناستوموز مارتین-گروبر نامیده می‌شود، توسط مطالعات آناتومیک و یا الکترودیاگنوستیک قابل اثبات است. دانش این آناستوموز از اهمیت ویژه‌ای در ضایعات محیطی اعصاب مدیان و اولنار برخوردار است. بنابراین در قدم اول -به‌عنوان هدف اصلی- شیوع این آناستوموز در جوامع مختلف باید تعیین گردد. مطالعه اخیر در بیمارستان شهدای تجریش در سال 1381 انجام شده است. روش بررسی: مطالعه در یک گروه 106 نفری (212 دست) که از نظر اعصاب محیطی دست‌ها کاملاً سالم بودند، انجام گرفت و افراد دارای بیماری‌های CTS، نوروپاتی اولنار و پلی‌نوروپاتی از مطالعه حذف شدند. تحریک Supramaximal روی اعصاب مدیان و اولنار در قسمت‌های پروگزیمال و دیستال داده و CAMP از عضلات A.P.B و A.D.M و F.D.I ثبت شد. کرایتریای تشخیصی آناستوموز شامل: 1- ارتفاع موج CAMP از عضله A.P.B و تحریک مدیان در قسمت پروگزیمال&le‌دیستال؛ 2- ارتفاع موج CAMP از عضله A.D.M و تحریک اولنار در قسمت دیستال‌<‌پروگزیمال؛ 3- ثبت negative CAMP از عضلات F.D.I یا A.D.M و با تحریک عصب مدیان در آرنج. یافته‌ها: در این مطالعه 212 بازو (50 درصد راست و 50 درصد چپ) مورد بررسی قرار گرفت. متوسط سنی بیماران 26 سال بود و شامل محدوده‌ای بین 15 تا 45 سال می‌شد و 52 نفر از بیماران زن بودند. 11 نفر از 106 بیمار مورد مطالعه براساس معیارهای تشخیصی، دارای آناستوموز بودند که شیوع 3/10 درصد را شامل می‌شود. نسبت زن به مرد نیز 4/7 است. نتیجه‌گیری: براساس این مطالعه شیوع آناستوموز حدوداً 10 درصد است؛ در‌حالی‌که در آخرین مطالعات دیگر شیوع آن 34-31 درصد گزارش شده است که برای اثبات آن لازم است مطالعات دیگری در سطح وسیع‌تری از جامعه به انجام برسد.

Title: تشخیص بیوشیمیایی و تعیین جهش‌های شایع در بیماری گالاکتوزمی

http://rehabilitationj.uswr.ac.ir/article-1-95-fa.html

هدف: گالاکتوزمی از بیماری‌های متابولیکی مادرزادی است که با الگوی وراثتی اتوزوم مغلوب به ارث می‌رسد و در اثر فقدان یا کاهش فعالیت آنزیم گالاکتوز فسفات یوریدیل ترانسفراز (GALT EC 2.7.7.12) ایجاد می‌شود. هدف از این تحقیق، تشخیص بیوشیمیایی و ملکولی بیماری گالاکتوزمی و تعیین شایع‌ترین موتاسیون ایجاد‌کننده بیماری بوده است که برای اولین‌بار در ایران انجام شده است. روش بررسی: در این مطالعه تشخیص بیوشیمیایی گالاکتوزمی با استفاده از سنجش کیفی فعالیت آنزیم GALT در گلبول‌های قرمز به روش Beutler برای 135 خانواده -که توسط پزشکان مشکوک به بیماری گالاکتوزمی بودند- انجام گرفت. در مجموع 16 خانواده، نقص در آنزیم GALT داشتند. سپس خانواده‌هایی که گالاکتوزمی در آنها قطعی شده بود برای جهش‌های Q188R ، K285N، L195P ، X380R و Q169K (معروف‌ترین جهش‌های عامل بیماری) به روش PCR-RFLP بررسی شدند. یافته‌ها: در مجموع ۱۶ خانواده نقص در آنزیم GALT داشتند. 8 خانواده جهش Q188R را نشان دادند و یک بیمار هتروزیگوت مرکب برای جهش K285N یافت شد. جهش‌های L195P، X380R و Q169K در هیچ یک از خانواده‌ها شناسایی نشد. نتیجه‌گیری: راه‌اندازی تشخیص بیوشیمیایی بیماری گالاکتوزمیا در بیمارستان‌های بزرگ اطفال بسیار ضروری و حیاتی است. همچنین بیماری گالاکتوزمیا از نظر ملکولی یک بیماری هتروژن است و جهش‌های ژنتیکی مختلفی می‌تواند در ابتلا به این بیماری مؤثر باشد که از بین آنها جهش Q188R شایع‌ترین جهش به‌شمار می‌آید.

Title: عوامل تسهیل‌کننده و موانع ارائه خدمات سلامتی در زمان وقوع بلایای طبیعی

http://rehabilitationj.uswr.ac.ir/article-1-96-fa.html

هدف: بحران‌های ناشی از بلایای طبیعی، به‌علت قطع روند طبیعی زندگی، آثار ناگواری مانند مرگ‌و‌میر، جراحت، ازکارافتادگی، تخریب تشکیلات اجتماعی و ایجاد حجم زیادی از نیازهای انسانی، مانند کمک‌های اولیه و خدمات پزشکی، نیازمند مدیریتی توانمند و هماهنگ در سطوح مسئولین و نهادهای اجرایی است. در مراحل اولیه بلایا، خدمات سلامتی عامل اصلی بقا هستند. ازآنجایی‌که بلایا تأثیر چشمگیری بر بهداشت، سلامت عمومی و رفاه جامعه آسیب ‌دیده می‌گذارد؛ لذا بررسی موانع و عوامل تسهیل‌کننده خدمات سلامتی در زمان وقوع بلایا بسیار حائز اهمیت است. در این مقاله سعی شده است براساس تجربیات ارائه‌دهندگان خدمات سلامتی در بلایا به بررسی عوامل بازدارنده و تسهیل‌کننده در ارائه خدمات سلامتی در زمان وقوع بلایا پرداخته شود. روش بررسی: پژوهش حاضر به روش گراندد تئوری انجام شد. نمونه‌ها 14 نفر شامل 4 پرستار، روان‌پزشک، مددکار، روانشناس، پزشک عمومی، کارشناس بهداشت، اپیدمیولوژیست و دو نفر از اهالی بم بودند. برای جمع‌آوری داده‌ها از مصاحبه‌های نیمه‌ساختار‌یافته استفاده شد. کلیه مصاحبه‌ها ضبط و سپس کلمه‌به‌کلمه دست‌نویس شد تا مورد تجزیه و تحلیل قرار گیرد. تجزیه و تحلیل داده‌های این تحقیق به روش استراوس و کوربین صورت گرفت. یافته‌ها: عوامل انسانی، هماهنگی، امکانات و تجهیزات، مشکلات ‌مدیریتی و فرهنگی و اطلاعاتی با کدهایی مثل کمبود نیروی متخصص، عدم هماهنگی، کمبود نیرو و تجهیزات، خودمحوری، نبود اطلاع‌رسانی و... به‌عنوان موانع و عوامل بازدارنده ارائه خدمات سلامتی توسط مشارکت‌کنندگان مطرح شدند. عوامل تسهیل‌کننده ارائه خدمات سلامتی شامل مدیریت، خدمات فرهنگی-آموزشی، نیروی انسانی و امکانات و تجهیزات با کدهای مدیریت واحد، لزوم هماهنگی، اهمیت حمل مصدوم، لزوم توجه به مسایل فرهنگی، لزوم وجود پزشک و پرستار، تأمین نیازها براساس فرهنگ و ... توسط مشارکت‌کنندگان مطرح شدند. نتیجه‌گیری: شرکت‌کنندگان در این مطالعه معتقدند که بزرگترین مانع برای ارائه خدمات سلامتی در زمان وقوع بلایا عدم هماهنگی است که در عواملی مثل مدیریت، نیروی انسانی، تجهیزات و اطلاع‌رسانی به‌طور کامل مشهود بود و می‌توان با هماهنگی کاری و طی فرایندی شامل بررسی سریع محل، فراخوان و اعزام گروه‌ها، تأمین و توزیع اطلاعات و ارزیابی مداوم عملکرد خدمات کارآ، سریع و با کیفیت بالا به مردم منطقه آسیب‌دیده ارائه داد.

Title: بررسی متغیرهای خانوادگی و فردی در افراد در معرض خطر سوء‌مصرف مواد

http://rehabilitationj.uswr.ac.ir/article-1-97-fa.html

هدف: بنابر پژوهش‌های انجام‌شده در زمینه علل گرایش افراد به مصرف و سوءمصرف مواد مخدر، این پژوهش بر آن است تا افراد در معرض خطر سوء‌مصرف مواد مخدر را با توجه به متغیرهای خانوادگی و فردی مورد بررسی قرار دهد. روش بررسی: این مطالعه روی 716 نفر از افراد در معرض خطر سوء‌مصرف مواد مخدر در 11 شهرستان استان فارس انجام شد. ابزارهای پژوهش عبارت بودند از: 1- آزمون کانون کنترل راتر؛ 2- پرسشنامه سبک‌های دلبستگی؛ 3- پرسشنامه پیوند والدینی؛ 4- مقیاس تاب‌آوری کونور و دیویدسون؛ 5- پرسشنامه مهارت‌های مقابله‌ای وین‌تراب؛ 6- پرسشنامه عزت‌نفس کوپر-اسمیت. یافته‌ها: تمامی متغیرها بین گروه سالم و گروه‌های مصرف‌کننده و سوء‌ مصرف‌کننده تفاوت معنی‌دار داشتند؛ بدین‌صورت که در متغیرهای تاب‌آوری، اعتمادبه‌نفس، مهارت‌های مسئله‌مدار، مراقبت و سبک دلبستگی ایمن، نمرات گروه سالم بالاتر از نمرات گروه‌های مصرف‌کننده و سوء مصرف‌کننده بود. اما در متغیر محافظت بیش‌ازحد، سبک دلبستگی دوسوگرا، کانون کنترل (نمره بالا به‌معنی کانون کنترل بیرونی)، تطابق هیجان‌مدار و تطابق کمتر مفید و غیر‌مؤثر نمرات گروه سالم کمتر از گروه‌های مصرف‌کننده و سوء مصرف‌کننده بود. مقایسه گروه‌های مصرف‌کننده و سوء مصرف‌کننده نشان داد که در متغیرهای تاب‌آوری، دوسوگرایی، مهارت‌های مشکل‌مدار و غیرمؤثر گروه‌های مصرف‌کننده و سوء مصرف‌کننده با یکدیگر تفاوت معنی‌دار داشتند. اما در متغیرهای مراقبت، محافظت بیش‌ازحد و سبک دلبستگی ایمن، اعتماد‌به‌نفس، کانون کنترل و تطابق هیجان‌مدار تفاوت بین دو گروه معنی‌دار نبود. نتیجه‌گیری: براساس یافته‌های پژوهش حاضر، جهت افزایش تاب‌آوری در برابر سوء‌مصرف مواد مخدر، لازم است تا مداخلات پیشگیرانه در سال‌های اولیه عمر کودک از طریق آموزش والدین در زمینه مراقبت و محافظت کافی و تلاش برای شکل‌دهی سبک دلبستگی ایمن، راهبردهای ایجاد اعتماد‌به‌نفس، کانون کنترل درونی و به‌کارگیری مهارت تطابقی مشکل‌مدار متمرکز باشند. این یافته‌ها همچنین تلویحاتی را برای مداخلات روان‌شناختی ارائه می‌دهد.

Title: بررسی و مقایسه برخی از جنبه‌های مهارت واج‌شناختی در دو روش کل‌خوانی

http://rehabilitationj.uswr.ac.ir/article-1-98-fa.html

هدف: آگاهی واج‌شناختی یکی از مهارت‌های فرازبانی است و به آگاهی فرد از صداهای سازنده کلمه اطلاق می‌گردد. این توانایی یکی از پیش‌نیازهای ضروری برای یادگیری و کسب مهارت در خواندن محسوب می‌شود و بسیاری از محققان به وجود رابطه‌ای دوسویه بین این مهارت و خواندن اشاره دارند. در پژوهش حاضر، سطح آگاهی واج‌شناختی در دانش‌آموزان دختر طبیعی پایه اول شهرستان‌های اصفهان و شاهین‌شهر که به دو شیوه مرسوم یادگیری خواندن در سیستم آموزشی ایران-روش کل خوانی و روش آوایی-تحت آموزش قرار گرفته بودند، تعیین گردید.  روش بررسی: این پژوهش مقطعی بوده و به شیوه توصیفی- تحلیلی برروی 106 نفر از دانش آموزان دختر مدارس ابتدایی شهرستانهای اصفهان و شاهین شهرکه به صورت تصادفی انتخاب شده بودند، صورت پذیرفت که تعداد 51 نفر از این دانش آموزان به شیوه آوایی و بقیه یعنی 55 نفر دیگر به شیوه کل خوانی آموزش دیده بودند.نحوه انتخاب آزمودنی ها به این صورت بود که پس از حصول اطمینان از اینکه این دانش آموزان مبتلا به اختلات گویایی، مشکلات بینایی، اختلالات خواندن، اختلالات یادگیری واعصاب مغزی کرانیال نبودند تحت آزمون آگاهی واجشناختی قرار می گرفتند وپس از انجام آزمون، امتیازات کودکان ثبت می گردید.در نهایت تجزیه و تحلیل اطلاعات با استفاده از نرم افزار Spss و آزمون t صورت پذیرفت. یافته‌ها: میانگین امتیازات در آزمون آگاهی واجشناختی در دو روش آوایی و کل خوانی در کودکان دختر پایه اول به ترتیب عبارت بود از 51/98 و 57/46 . تفاوت میانگین این دو گروه به لحاظ آماری معنادار می‌باشد (0/047=P). همچنین از میان خرده آزمون‌های آگاهی واجشناختی امتیاز میانگین خرده آزمون حذف واج آغازین در دو روش خواندن (کل خوانی وآوایی) به ترتیب شامل 84/4 و 33/3 بود که حاکی از معنادار بودن تفاوت بین میانگین‌ها داشت (P=0/001). در بقیه خرده آزمون ها تفاوت معناداری مشاهده نگردید. نتیجه‌گیری: نتایجی که در این پژوهش کسب شد نشان داد بین میانگین امتیاز کل آگاهی واجشناختی با روش خواندن ارتباط معناداری وجود دارد و همچنین از بین خرده آزمون های آگاهی واجشناختی فقط بین امتیاز حذف واج آغازین در دو روش کل خوانی وآوایی تفاوت معناداری وجود دارد. به علت اهمیت بالای آگاهی واجشناختی به عنوان پیش بینی کننده توانایی خواندن در سالهای بعد لزوم انجام تحقیقات بیشتر از این دست بسیار احساس می‌گردد.

Title: نقش باورهای مذهبی درونی و بیرونی در سلامت روان و میزان افسردگی سالمندان

http://rehabilitationj.uswr.ac.ir/article-1-99-fa.html

هدف : هدف از پژوهش حاضر بررسی نقش جهت گیری مذهبی( درونی و بیرونی) در سلامت روان و میزان افسردگی سالمندان مقیم مراکز بهزیستی و مقیم جامعه بود. روش بررسی: روش مطالعه از نوع مطالعات پس رویدادی و همبستگی است که با روش نمونه گیری تصادفی و خوشه ای تعداد 230 آزمودنی از افراد سالمند در مراکز سالمندی تحت پوشش سازمان بهزیستی و مکانهای عمومی (مسجد و  پارک) که محل تجمع سالمندان مقیم جامعه است از دو جنس زن و مرد انتخاب و آزمودنیها ابتدا از نظر اختلالات شناختی سرند گردیدند.سپس آزمونهای 28 سوالی سلامت عمومی، افسردگی بک و آزمون نگرش مذهبی آلپورت تکمیل و با استفاده از آزمونهای آماری همبستگی پیرسون و من ویتنی نتایج مورد تجزیه و تحلیل قرار گرفت. یافته‌ها: نتایج نشان داد بین جهت گیری مذهبی با سلامت روان و افسردگی سالمندان رابطه همبستگی معنادار وجود دارد، بدین معنا که هر چه نمرات جهت گیری مذهبی به جهت بیرونی افزایش می‌یابد، نمرات اختلال در سلامت روان و افسردگی بالا می‌رود و هر چه نمرات به سمت جهت گیری مذهب درونی تمایل می‌یابد، نمرات اختلال در سلامت روان و افسردگی کاهش می‌یابد. همچنین بین سلامت روان، افسردگی و جهت گیری مذهبی سالمندان مقیم و غیر مقیم جامعه تفاوت معنادار وجود دارد. بدین معنا که افراد سالمند مقیم مراکز از جهت گیری مذهبی بیرونی تربرخوردار و اختلال در سلامت روانی و میزان افسردگی بالاتری را نسبت به گروه سالمندان مقیم جامعه دارند. نتیجه‌گیری: باور مذهب بیرونی با اختلال در سلامت روان و افسردگی و باور مذهب درونی با سلامت روان رابطه دارند. همچنین اختلال روانی و افسردگی در بین سالمندان مقیم بیشتر از غیر مقیم جامعه است ضمن آنکه سالمندان مقیم دارای نگرش مذهب بیرونی تر هستند.

Title: ژنتیک ناشنوایی

http://rehabilitationj.uswr.ac.ir/article-1-100-fa.html

از هر 1000 موالید زنده یک نفر مبتلا به ناشنوایی عمیق می‌باشد که در %50 از این افراد علت ژنتیکی به عنوان منشاء این بیماری مشخص شده است و در %50 دیگر علل اکتسابی می‌باشد. تاکنون بیش از 350 شرایط ژنتیکی مختلف که موجب ناشنوایی می‌شوند، گزارش شده است که در بین آنها %70 باعث ایجاد ناشنوایی غیر سندرمی و %30 دیگر باعث ایجاد ناشنوایی سندرمی می‌شوند. انواع غیر سندرمی و سندرمی ناشنوایی می‌توانند به چهار شکل جسمی غالب (DFNA)، جسمی مغلوب (DFNB)، وابسته به ایکس (DFN) و میتوکندریایی باشند. در این میان %80-75 از ناشنوایی‌های با علت ژنتیکی بصورت اتوزومی مغلوب ، %20-10 به صورت اتوزومی غالب و %5-1 وابسته به X یا با علت میتوکندریایی %2-0 می‌باشند تا کنون 51 جایگاه برای ناشنوایی اتوزومال غالب و 61 جایگاه برای اتوزومی مغلوب و 7 جایگاه برای وابسته به X شناسایی شده است. ناشنوایی غیر سندرمی با توجه به سن شروع بیماری به دو دسته پیش کلامی و پس کلامی تقسیم بندی می‌شود. بعنوان یک قانون کلی در بیشتر ناشنوایی‌های غیر سندرمی با الگوی توارث جسمی غالب اختلال در ناشنوایی در سنین بالاتر و به صورت پس کلامی دیده می‌شود، حال آنکه در ناشنوایی‌های غیر سندرمی جسمی مغلوب،‌ ناشنوایی معمولاً پیش کلامی می‌باشد.
